# Efficient reduction of vanadium (V) with biochar and experimental parameters optimized by response surface methodology

**DOI:** 10.1038/s41598-024-58880-4

**Published:** 2024-04-06

**Authors:** Hao Peng, Laixin Wang, Jing Guo, Yuting Wu, Bing Li, Yinhe Lin

**Affiliations:** 1 Chongqing Key Laboratory of Inorganic Special Functional Materials, College of Chemistry and Chemical Engineering, Yangtze Normal University, Fuling, Chongqing 408100 People’s Republic of China; 2 Intelligent Development Department, Huatian Engineering & Technology Corporation, MCC, Nanjing, Anhui People’s Republic of China; 3 College of Chemistry and Chemical Engineering, Chongqing University, Chongqing, 408100 People’s Republic of China

**Keywords:** Vanadium, Response surface methodology, Reduction, Biochar, Thermodynamics, Chemistry, Environmental chemistry, Pollution remediation

## Abstract

Water pollution deteriorates ecosystems and has a great threaten to the environment. The environmental benefits of wastewater treatment are extremely important to minimize pollutants. Here, the biochar purchased from the related industry was used to treat the wastewater which contained high concentration of vanadium (V). The concentration of vanadium was measured by the IC-OES and the results showed that 96.1% vanadium (V) was reduced at selected reaction conditions: the mass ratio of biochar to vanadium of 5.4, reaction temperature of 90 °C, reaction time at 60 min and concentration of H_2_SO_4_ of 10 g/L, respectively. Response surface methodology confirmed that all the experimental parameters had positive effect on the reduction of vanadium (V), which could improve the reduction efficiency of vanadium (V) as increased. The influence of each parameter on the reduction process followed the order: A (Concentration of H_2_SO_4_) > C (mass ratio of biochar to vanadium) > B (mass ratio of biochar to vanadium). Especially, the mass ratio of biochar to vanadium and concentration of H_2_SO_4_ had the greatest influence on the reduction process. This paper provides a versatile strategy for the treatment of wastewater containing vanadium (V) and shows a bright tomorrow for wastewater treatment.

## Introduction

Vanadium, as one of the transition metals, is existed in multiple valences including II, III, IV, and V in the environment, among which, vanadium in IV, and V are stable and common^1–4^. Vanadium's low concentration is beneficial for healthy cell proliferation, while high concentration increases the risk of functional lesions in spleen, bones, liver, kidneys and nervous system by food chain^5–8^. Vanadium is belonged to the list of environmentally hazardous elements commented by the United Nations Environment Program (UNEP) and the limit level is set as below 0.2 μg/L and below 0.05 μg/L in China (Standard of China GB 5749-2006)^9–12^. Compared with other heavy metals, vanadium is more toxic due to its strong oxidative damage to the cells. Thus, it should be removed before wastewater discharge.

Many methods like biological remediation, adsorption and reduction had been developed for vanadium removal. Biological remediation gained attentions due to its low cost and potential applications for in-situ remediation^13–16^, but knowledge is limited on their interaction during the process as well as their biogeochemical cycling in groundwater. Another low cost and easy-operation technology is adsorption, which has been widely applied in treatment of heavy metal containing wastewater^17–19^. And many materials are evaluated, such as zeolite, chitosan, biochar, and orange peel^20–22^. However, adsorption is limited to the large scale and industrial application, also the low concentration of vanadium in the vanadium-containing water streams. Commonly reduction of vanadium (V) to vanadium (IV) is recognized as a feasible method to detoxify them in groundwater as the vanadium (IV) had less toxic and mobile^23^. Biochar derived from municipal solid waste, straw, wood, manure, sludge, and shell waste is a typical material used in the pollution control because of its low-cost and abundant feed stock availability^24–27^. In addition, the large surface area, high mineral content, and rich oxygen-containing functional groups of biochar were favorable for adsorption of wastewater contaminants such as antibiotics, dyes, and heavy metals. However, in our recent studies, biochar had been proved to be an efficient reductant for high valence heavy metal control^18,19,28,29^. Thus, biochar was applied to treat vanadium-containing wastewater in this paper. The experimental parameters including the mass ratio of biochar to vanadium (m(C)/m(V)), reaction temperature, reaction time and concentration of H_2_SO_4_ on the reduction process were investigated. In order to optimize the reaction conditions, the response surface methodology was also investigated.

## Materials and methods

### Materials

Sodium vanadate, sulfuric acid and biochar were of analytical grade and purchased from Kelong Co., Ltd, Chengdu, China. All solutions were prepared with deionized water with a resistivity greater than 18 MΩ/cm (HMC-WS10).

### Experimental procedure

The detailed experimental procedure could be seen in our previous works^30–34^. For the batch experiments, 0.05 M sodium vanadate solution (prepared by dissolving amount of sodium vanadate in the distilled water) was added into the 300 mL beaker placed in a water bath, the initial pH of the vanadium solution was adjusted by adding sulfuric acid. After the temperature was heated to the determined, the biochar was added and then stirred at 500 rpm. During the reaction process, the samples were collected every 5 min and the concentration of vanadium (V) were measured by Inductively Coupled Plasma Optical Emission Spectrometer (ICP-OES, Optima 5300DV)^31,32,34^, and the reduction efficiency (η) of vanadium (V) was calculated by following Eq. (1):

1 $$\upeta = \frac{{{\text{C}}{}_{{{\text{t}} - 1}} - {\text{C}}{}_{{\text{t}}}}}{{{\text{C}}{}_{{{\text{t}} - 1}}}} \times 100\%$$

where *C*_*t*_,* C*_t − 1_*,* are the concentration of vanadium (V) at reaction time of t and the last time, mg/L.

### Response surface methodology

The response surface methodology (RSM) was applied to optimize the experimental process and order the significance of experimental parameters as the single factor ignore the interactions between the parameters (seen in the supporting information)^25,32,35–37^. The whole design was conducted in the software Design Expert 8.0. The experimental parameters were set as A (Concentration of H_2_SO_4_), B (Reaction Temperature) and C (m(C)/m(V)), reduction efficiency was set as the response. The actual values for them were confirmed through the single factor experimental results and displayed in Table 1*.*

**Table 1:** Parameters and level values.

Parameters	Unit	Level		
		− 1	0	1
A: Concentration of H_2_SO_4_ B: Reaction temperature	–	0	15	30
	°C	30	60	90
C: (m(C)/m(V)		0.9	3.3	5.4

## Results and discussion

### Thermodynamics analysis

**Figure 1:**
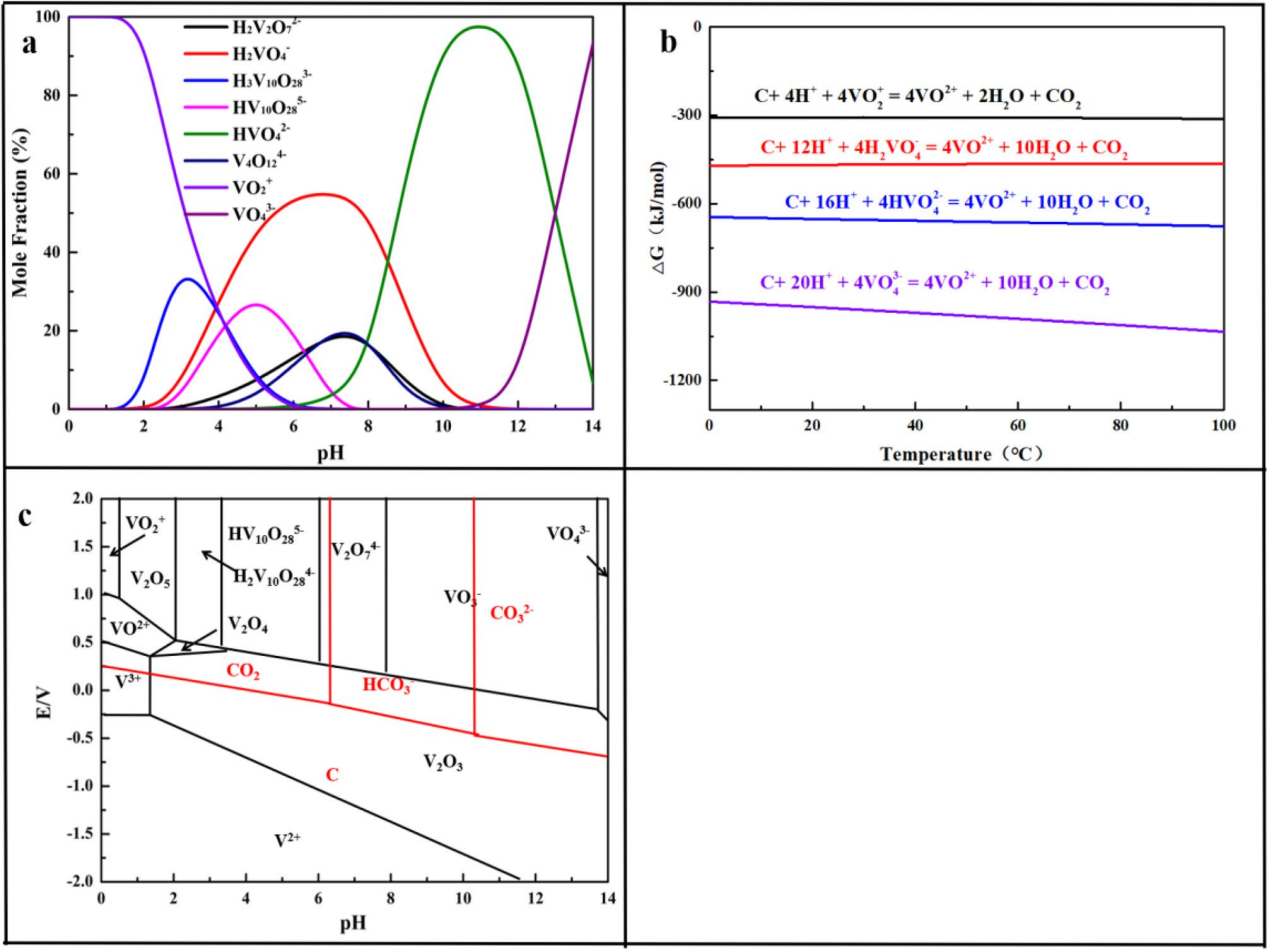
(**a**) vanadium species in vanadium (V)-H_2_O system; (**b**) relationship between ΔG and temperature of main reactions; (**c**) E-pH diagram of vanadium-chromium-manganese system at 25 °C.

Figure 1a summarized the mole distribution of vanadium species in the aqueous solution at [V] = 0.05 mol/L, the results indicated that the vanadium (V) existed in the form of VO_2_^+^, HVO_4_^2−^, H_2_VO_4_^−^, V_2_O_7_^4−^, HV_2_O_7_^3−^, H_2_V_2_O_7_^2−^, V_4_O_12_^4−^, V_4_O_13_^6−^, HV_4_O_13_^5−^, V_5_O_15_^5−^, V_6_O_18_^6−^, V_10_O_28_^6−^, HV_10_O_28_^5−^, H_2_V_10_O_28_^4−^, H_3_V_10_O_28_^3−^^18,31,38^. The main reactions during the reduction process were likely reacted as Eqs. (2), (3), (4) and (5). The standard Gibbs energy ($\Delta {\text{G}}_{{\text{T}}}^{\uptheta }$) of these reaction equations at selected reaction temperatures could be calculated with $\Delta_{{\text{f}}} {\text{H}}_{{{298}}}^{\uptheta }$, ${\text{S}}_{{{298}}}^{\uptheta }$ and ${\text{C}}_{{\text{p}}}$ following Eqs. (6), (7) and (8)^39,40^.

2 $${\text{C}} + 4{\text{H}}^{ + } + 4{\text{VO}}_{2}^{ + } = 4{\text{VO}}^{2 + } + 2{\text{H}}_{{2}} {\text{O}} + {\text{CO}}_{{2}}$$

3 $${\text{C}} + 12{\text{H}}^{ + } + 4{\text{H}}_{{2}} {\text{VO}}_{4}^{ - } = 4{\text{VO}}^{2 + } + 10{\text{H}}_{{2}} {\text{O}} + {\text{CO}}_{{2}}$$

4 $${\text{C}} + 16{\text{H}}^{ + } + 4{\text{HVO}}_{4}^{2 - } = 4{\text{VO}}^{2 + } + 10{\text{H}}_{{2}} {\text{O}} + {\text{CO}}_{{2}}$$

5 $${\text{C}} + 2{\text{0H}}^{ + } + 4{\text{VO}}_{4}^{3 - } = 4{\text{VO}}^{2 + } + 10{\text{H}}_{{2}} {\text{O}} + {\text{CO}}_{{2}}$$

6 $$\Delta {\text{G}}_{{\text{T}}}^{\uptheta } { = }\Delta {\text{H}}_{{\text{T}}}^{\uptheta } - {\text{T}}\Delta {\text{S}}_{{\text{T}}}^{\uptheta }$$

7 $$\Delta {\text{H}}_{{\text{T}}}^{\uptheta } = \Delta {\text{H}}_{{{298}}}^{\uptheta } { + }\int_{{{298}}}^{{\text{T}}} {\Delta {\text{C}}_{{\text{p}}} } {\text{dT}}$$

8 $$\Delta {\text{S}}_{{\text{T}}}^{\uptheta } = \Delta {\text{S}}_{{{298}}}^{\uptheta } { + }\int_{{{298}}}^{{\text{T}}} {\frac{{\Delta {\text{C}}_{{\text{p}}} }}{{\text{T}}}} {\text{dT}}$$

Equation (9) was obtained by merging Eqs. (6), (7) and (8).

9 $$\Delta {\text{G}}_{{\text{T}}}^{\uptheta } = \Delta {\text{H}}_{298}^{\uptheta } - {\text{T}}\Delta {\text{S}}_{298}^{\uptheta } + \int_{{{298}}}^{{\text{T}}} {\Delta {\text{C}}_{{\text{p}}} } {\text{dT}} - {\text{T}}\int_{{{298}}}^{{\text{T}}} {\frac{{\Delta {\text{C}}_{{\text{p}}} }}{{\text{T}}}} {\text{dT}}$$

And $\Delta {\text{Cp}}$ was calculated as Eq. (10).

10 $$\Delta {\text{C}}_{{\text{p}}} = \Delta {\text{a}} + \Delta {\text{b}} \times 10^{ - 3} {\text{T}} + \Delta {\text{c}} \times 10^{5} {\text{T}}^{ - 2} + \Delta {\text{d}} \times 10^{ - 6} {\text{T}}^{2}$$

Then $\Delta {\text{G}}_{{\text{T}}}^{\uptheta }$ was calculated as Eq. (11).

11 $$\Delta {\text{G}}_{{\text{T}}}^{\uptheta } = \Delta {\text{H}}_{298}^{\uptheta } - {\text{T}}\Delta {\text{S}}_{298}^{\uptheta } - {\text{T}}\int_{{{298}}}^{{\text{T}}} {\frac{{{\text{dT}}}}{{{\text{T}}^{2} }}} \int_{{{298}}}^{{\text{T}}} {(\Delta {\text{a}} + \Delta {\text{b}} \times 10^{ - 3} {\text{T}} + \Delta {\text{c}} \times 10^{5} {\text{T}}^{ - 2} + \Delta {\text{d}} \times 10^{ - 6} {\text{T}}^{2} )} {\text{ dT}}$$

Integrate.

12 $$\begin{aligned} \Delta {\text{G}}_{{\text{T}}}^{\uptheta } & { = }\Delta {\text{H}}_{298}^{\uptheta } - {\text{T}}\Delta {\text{S}}_{298}^{\uptheta } - T\left\{ {\Delta {\text{a}}\left( {\ln \frac{{\text{T}}}{298} + \frac{298}{{\text{T}}} - 1} \right){ + }\Delta {\text{b}} \times 10^{ - 3} \left[ {\frac{1}{{2{\text{T}}}}({\text{T}} - 298)^{2} } \right]} \right. \\ & \quad \left. { + \frac{{\Delta {\text{c}} \times 10^{5} }}{2}\left( {\frac{1}{298} - \frac{1}{{\text{T}}}} \right)^{2} + \Delta {\text{d}} \times 10^{ - 6} \left( {\frac{{{\text{T}}^{2} }}{6} + \frac{{298^{3} }}{3T} - \frac{{298^{2} }}{2}} \right)} \right\} \\ \end{aligned}$$

The $\Delta_{{\text{f}}} {\text{H}}_{{{298}}}^{\uptheta }$, $\Delta {\text{S}}_{{\text{T}}}^{\uptheta }$, *a, b, c* and *d* in Eq. (12) could be obtained from the handbook.

The results showed in Fig. 1b displayed that the ΔG of the Eqs. (2)–(5) which occurred during the reduction process were all negative at selective reaction temperatures. It was concluded that the reduction process was easy to occur in thermodynamics. The E-pH diagram of vanadium and biochar measured by HSC Chemistry 6.0 was shown in Fig. 1c, it was clear that vanadium (V) was above than biochar, which meant that the oxidation–reduction potential of vanadium (V) was higher than biochar, thus, the biochar could be used as a reductant to reduce vanadium (V) into vanadium (IV) in theory.

### Reduction process

**Figure 2:**
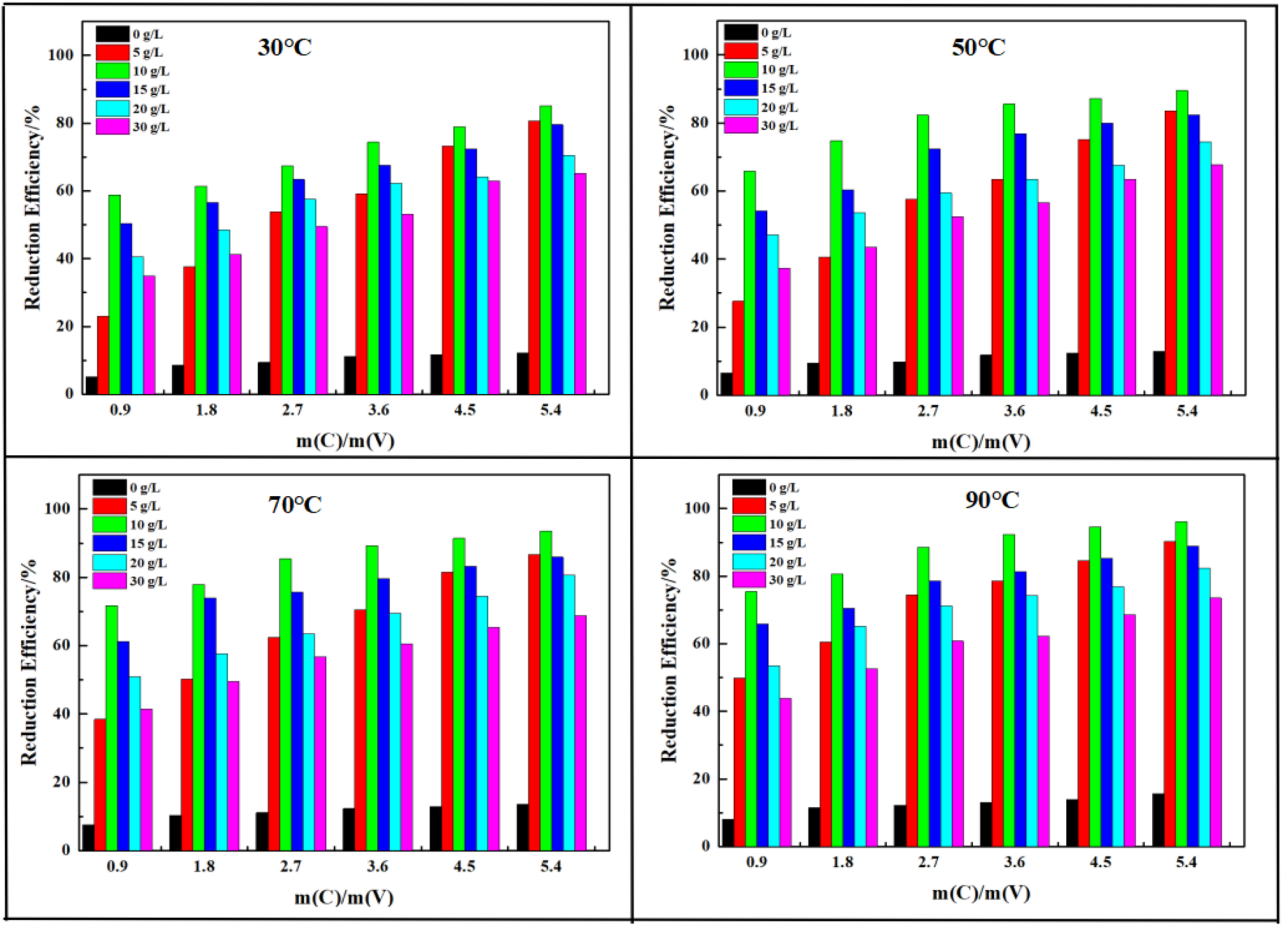
Effect of the single parameters on the reduction process.

In this paper, the effect of parameters including the mass ratio of biochar to vanadium (m(C)/m(V)), reaction temperature, reaction time and concentration of H_2_SO_4_ on the reduction process were investigated, the results were displayed in Fig. 2.

The mass ratio of biochar to vanadium had a significant effect on the reduction of vanadium (V) as it was the main reaction reagent. A series of experiments were conducted to investigate the effect of the mass ratio of biochar to vanadium (m(C)/m(V)) on the reduction process. The m(C)/m(V) was set as m(C)/m(V) = 0.9, 1.8, 2.7, 3.6, 4.5 and 5.4, respectively. The results showed that the reduce efficiency of vanadium was increased with the increase of m(C)/m(V) at selected reaction temperatures. High dosage was beneficial for the reduction process as the biochar was the direct reaction reagent. The reduction efficiency of vanadium was just 5.2% at m(C)/m(V) = 0.9, concentration of H_2_SO_4_ was 0 g/L and reaction temperature of 30 °C, it was improved to 12.1% at the m(C)/m(V) = 0.9 while other conditions were kept. It was just increased 7 percentages. While at concentration of H_2_SO_4_ was 10 g/L, the reduction efficiency was increased from 58.8% to 85.1% as mass ratio of biochar to vanadium increased from m(C)/m(V) = 0.9 to m(C)/m(V) = 5.4. The large improvement indicated that the mass ratio of biochar to vanadium had significant effect on the reduction process.

Usually, reaction temperature played an important role in a standard chemical reaction. In this paper, reaction temperature was set as 30, 50, 70, and 90 °C, respectively. Compared the experimental results at selected temperatures, the reduction efficiency was increased with the increasing of reaction temperature, and the increasing trend of reduction efficiency was similar with mass ratio of biochar to vanadium, which indicated that both mass ratio of biochar to vanadium and reaction temperature had significant effect on the reduction process. Higher temperature could intensify the activity of biochar molecule and vanadium (V) ion, promote the extent of the reduction reaction and enforce the reduction of vanadium (V). The maximum reduction efficiency of vanadium at reaction temperature of 30 °C was 85.1% at m(C)/m(V) = 5.4 with 10 g/L H_2_SO_4_, while at the reaction temperature of 90 °C, the reduction efficiency was 90.2% just at m(C)/m(V) = 0.9 and increased to 96.1% at at m(C)/m(V) = 5.4. The results indicated that the higher reaction temperature could enhance the reduction process significantly.

The results showed in Fig. 2 indicated that the concentration of H_2_SO_4_ had different effect on the reduction efficiency with mass ratio of biochar to vanadium and reaction temperature. When the concentration of H_2_SO_4_ was 0 g/L, the vanadium was existed as HVO_4_^2−^ and VO_4_^3−^ owing to the high alkaline solution, the reaction was not significant and little vanadium was reduced. With the increase of concentration of H_2_SO_4_, the existence of vanadium was changed, the maximum reduction efficiency was achieved at 10 g/L and then decreased follow the increase of concentration of H_2_SO_4_. The reduction reaction was simple while the experimental results were special and the detailed reaction mechanism between biochar with polymeric vanadium ions was not clear and needed further study in our future works. At concentration of H_2_SO_4_ was 0 g/L, the maximum reduction efficiency of vanadium was just 15.6% at m(C)/m(V) = 0.9 and reaction temperature of 90 °C, it was improved to 96.1% at concentration of H_2_SO_4_ was 10 g/L and decreased to 73.5% at concentration of H_2_SO_4_ was 30 g/L as other conditions kept.

As discussed above, the experimental parameters all had significant effect on the reduction process, in order to optimize the reaction conditions, response surface methodology was introduced.

### Response surface methodology

Response surface methodology was an efficient method which offered a large amount of information from a relative small number of experiments, allowing the observation of both the effect of the independent variables on the response as well as their possible interactions. And it had been extensively applied for optimization study of test parameters and obtained response surfaces^25,32,35–37^. From the results analyzed above, the parameters including the mass ratio of biochar to vanadium (m(C)/m(V)), reaction temperature, reaction time and concentration of H_2_SO_4_ all had influences on the reduction process, but was hard to distinguish the important for all. Thus, the response surface methodology was applied.

#### Model fitting

The squares root was used to express the simulated results and it was presented in Eq. (13):

$${\text{sqrt }}\left(\upeta \right) = {8}.{77} + {2}.{21}*{\text{A}} + 0.{3}0*{\text{B}} + 0.{63}*{\text{C}} + 0.0{82}*{\text{AB}} - 0.0{39}*{\text{AC}} - 0.{13}*{\text{BC}} - {3}.{1}0*{\text{A}}^{{2}} - 0.{14}*{\text{B}}^{{2}} - 0.{24}*{\text{C}}^{{2}}$$ (13)

**Figure 3:**
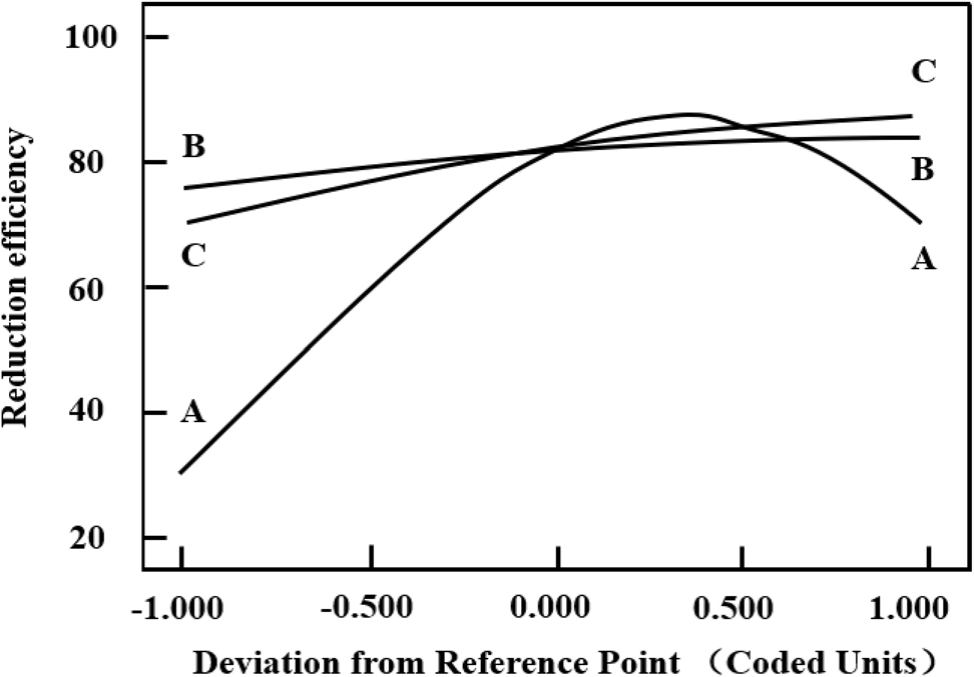
Perturbation plot for the reduction efficiency of V (V) in the design space (**A** Concentration of H_2_SO_4_, **B** reaction temperature, **C** (m(C)/m(V)).

The influence of each parameter on the reduction efficiency of vanadium (V) could be seen from the coefficients before them in the Eq. (13). The coefficients of them were 2.21, 0.30 and 0.63, respectively, which confirmed that all the parameters had positive effects on the reduction efficiency. The results displayed in Fig. 3 indicated that the influence of each parameter on the reduction efficiency followed the order: A > C > B, which was consistent with the results described in Eq. (13). Above all, the mass ratio of biochar to vanadium and concentration of H_2_SO_4_ had the greatest influence on the reduction process.

#### Response surface analysis

The reduction process of vanadium using biochar through various variables could be investigated through these model equations. Different parameters R^2^, P-values, F-values and adjusted R^2^ values were measured as standard that were helpful to determine the accuracy of every coefficient in order to appraise the significance of predicated model^35–37,41,42^. The ANOVA results (seen in Table 2) confirmed that model F-values of 148.66 showed that predicated model was substantial. There was only 0.01% chance that an F-values, which could occur owing to noise. The model P-values (< 0.0001) less than 0.050 indicted model terms were significant. In this optimization case, several model terms such as A, B, C and A^2^ were in significant form due to their less P-value. The values larger than 0.10 means insignificant model terms. In this case, AB, AC, BC, and B^2^ were insignificant model terms. The R^2^-value exhibited a measure of how much variability in the observed response values could be expressed by the experimental factors as well as their interactions by establishing a relationship between predicated and experimental consequences. R^2^ close to one revealed good fitting of experimental data into predicated model equation. The regression model produced higher R^2^ up to 0.9948 signifying excellent fitting between model as well as experimental data values. The Rredicated-R^2^ up to 0.9167 was in reasonable agreement with the Adjusted-R^2^ of 0.9881. The Adequate-precision was helpful to evaluate the signal-to-noise ratio. A ratio greater than 4 was desirable. Here, higher Adequate-precision of 34.441 revealed an adequate signal. This regression model could be applied to navigate the design space.

**Table 2:** Analysis of variance for the response.

Source	Sum of squares	Z	Mean square	F value	*p* value Prob > F
Model	84.54	9	9.39	148.66	< 0.0001
A	39.06	1	39.06	618.12	< 0.0001
B	0.72	1	0.72	11.37	0.0119
C	3.17	1	3.17	50.13	0.0002
AB	0.027	1	0.027	0.42	0.5362
AC	0.006099	1	0.006099	0.097	0.7651
BC	0.063	1	0.063	0.99	0.3531
A^2^	40.37	1	40.37	638.82	< 0.0001
B^2^	0.080	1	0.080	1.27	0.2968
C^2^	0.25	1	0.25	3.91	0.0886
Residual	0.44	7	0.063	–	–
Lack-of-fit	0.44	3	0.15	–	–
Pure error	0.000	4	0.000		

**Figure 4:**
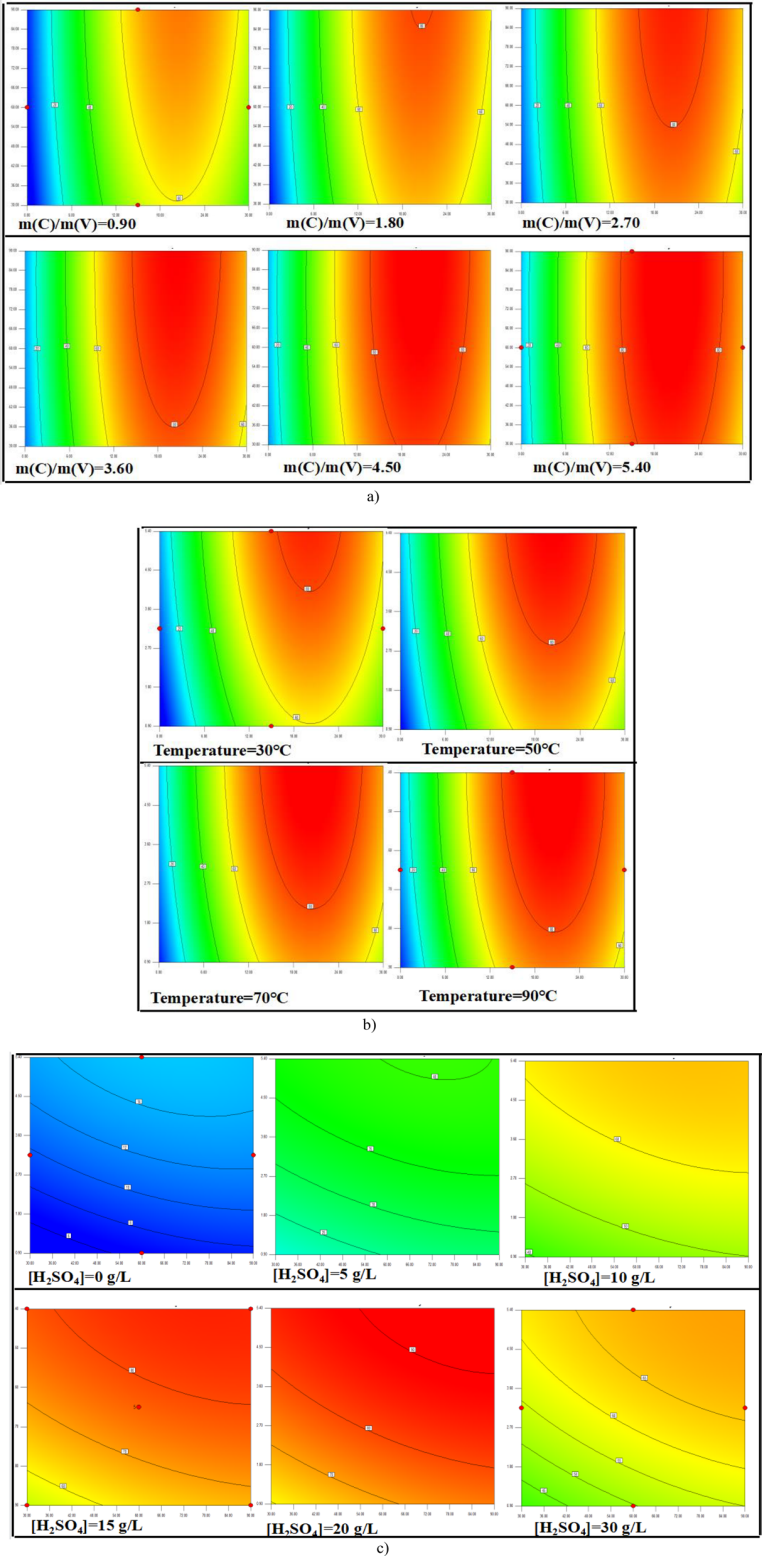
Response surface plots for factors. (**a**) X1 = A: [H_2_SO_4_], X2 = B: Temperature C: m(C)/m(V) = 0.90, 1.80, 2.70, 3.60, 4.50, 5.40. (**b**) X1 = A: [H_2_SO_4_], X2 = C: m(C)/m(V) B: Temperature = 30°C, 50°C, 70°C, 90°C. (**c**) X1 = B: Temperature, X2 = C: m(C)/m(V) A: [H_2_SO_4_] = 0 g/L, 5 g/L, 10 g/L, 15 g/L, 20 g/L, 30 g/L.

The possible inspiration of variables over maximum reduction efficiency for vanadium using biochar was explained through response surface plots. These response surface plots were helpful for the determination of cooperative association between specific parameter and response for the maximum reduction efficiency. Figure 4 described the 2-D counter plots of combined influence of four experimental parameters over the reduction efficiency for vanadium using biochar. The counter plots were providing the mutual interactions among the independent parameters. It could be analyzed that all selected parameters had direct effect over the reduction of vanadium. These response surface plots confirmed the perfect and strong interactions among the selected independent experimental parameters. Higher reaction temperature and higher mass ratio of biochar to vanadium were beneficial for the reduction process, the results were consistent with our previous studies^28,34^.

## Conclusions

A highly efficient reduction process of vanadium (V) with biochar was investigated and the following conclusions could be obtained:

1. The vanadium (V) was easily being reduced by biochar at high reaction temperature with high mass ratio of biochar to vanadium in acidic medium. Nearly 96.1% vanadium (V) was reduced at selected reaction conditions: the mass ratio of biochar to vanadium at m (C)/m(V) = 5.4, reaction temperature of 90 °C, reaction time at 60 min and concentration of H_2_SO_4_ of 10 g/L, respectively.
2. Response surface methodology confirmed that all the experimental parameters had positive effect on the reduction of vanadium (V). The influence of each parameter on the reduction process followed the order: A (concentration of H_2_SO_4_) > C (mass ratio of biochar to vanadium) < B (m (C)/m(V). Especially, the mass ratio of biochar to vanadium and concentration of H_2_SO_4_ had the greatest influence on the reduction process.

## Supplementary Information

Supplementary Information.

## Data Availability

All data generated or analyzed during this study are included in this published article and supporting information.
