# Supplementary material for: Efficient reduction of vanadium (V) with biochar and experimental parameters optimized by response surface methodology
Source: Sci Rep. 2024 Apr 6;14:8118. doi: 10.1038/s41598-024-58880-4 (PMC11372122; doi:10.1038/s41598-024-58880-4)
Supplement: Supplementary file 1 — Supplementary Information. [file 41598_2024_58880_MOESM1_ESM.docx]

Efficient Reduction of V (V) with Biochar and Parameters Optimized by Response Surface Methodology

Hao Peng ^※,1^, LaixinWang ^2^, Jing Guo ^3^, Yuting Wu ^1^, Bing Li ^1^, Yinhe Lin ^1^

1. Chongqing Key Laboratory of Inorganic Special Functional Materials, College of Chemistry and Chemical Engineering, Yangtze Normal University, Fuling, Chongqing 408100, P. R. China
2. Intelligent Development Department, Huatian Engineering & Technology Corporation, MCC, Anhui, China
3. College of Chemistry and Chemical Engineering, Chongqing University, Chongqing 408100, P. R. China

Corresponding author：[cqupenghao@126.com](mailto:cqupenghao@126.com) (Hao Peng)

**Response surface methodology**

The adoption of RSM to optimize a known process is easy practica1 and economica1. Successful RSM optimizations usually consist of three steps．The first step was to design appropriate experiments to efficiently assess the model parameters. The second step was to develop a polynomial model that can be applied to the experimental data through regression and to verify the model’s suitability by applying a statistical test (e.g., *lack-of-fit* or *F-test*). The final step was to determine the values of factors that result in the best conditions. A first or second-order polynomial was usually used for RSM analysis, and a second order polynomial was preferred for responses that include a curvature. The general form of such a polynomial is as follows：

$\text{y=a}_{0}\text{+}\sum_{\text{i=1}}^{\text{k}} \text{a}_{\text{i}}\text{x}_{\text{i}}+\sum_{\text{i=1}}^{\text{k}} \text{a}_{\text{ii}}\text{x}_{\text{i}}^{2}+\sum_{\text{i=1}}^{\text{k}} \sum_{\text{j=}1}^{\text{k}} \text{a}_{\text{ij}}\text{x}_{\text{i}}\text{x}_{\text{j}}^{2}+\varepsilon(\text{i<j)}$ (2)

Where*y* is the predicted response, *a_0_* is a constant, *a_i_*is the *i* th linear coefficient, *a_ii_* is the *i* th quadratic coefficient, *a_ij_* is the *i* th interaction coefficient, *x_i_* is an independent variable, *k*is the number of factors, and *ε* is the associated error.

The coefficients of the model were predicted using regression. Details of the parameter estimations for such a model have been reported previously. Central composite design (CCD), which was utilized in this study, was the most popular second-order experimental design and was an efficient approach to providing sufficient information to test the fitness of a model. The CCD approach did not require numerous design points; therefore, it saved the expense and time associated with completing experiments. Many experiments in which CCD had been applied had included three sets: (1) factional factorial runs (2^k-1^), which studied factors at -1 (minimum) and +1 (maximum) levels; (2) center-point runs, which examined factors at a center point of a design space and aided in the understanding of curvature and data replication to evaluate pure errors; and (3) axial or star-point runs (2k), which set al1 factors to 0 (i.e., the center point), except for one factor with values of +α and – α.

In this study, the Design-Expert software (Version 8.0.6) was used to design the experiments. CCD was applied to investigate the impact of process parameters on the reduction efficiency of vanadium. The experiment results were incorporated to determine an empirical equation that could predict the optimal operating conditions. In this paper, the experimental parameters were selected as A (initial pH of vanadium-containing wastewater), B (Reaction temperature), C (dosage of oxalic acid (n (O)/n(V)), and D (Reaction time). reduction efficiency was selected as the response. The satisfaction degree of the polynomial equation developed through a regression of Equation (2) was assessed on the basis of *R^2^* and *R_Adj_^2^. R^2^* was a measurement of the amount of variation around the mean, it was determined for a model using Equation (3). *R_Adj_^2^* was a measurement is a measurement of the amount of variation around the mean; it was determined by experiments and was regulated for the number of terms in the model using Equation (4).

$\text{R}^{2}\text{=1-}\frac{\text{S}_{\text{residual}}}{\text{S}_{\text{model}}\text{-S}_{\text{residual}}}$ (3)

$\text{R}_{\text{adj}}^{2}\text{=1-}\frac{\text{S}_{\text{residual}}/\text{Z}_{\text{residual}}}{\text{（S}_{\text{model}}\text{+S}_{\text{residual}}\text{）/(Z}_{\text{model}}\text{+Z}_{\text{residual}})}$ (4)

Where *S* is the sum of squares and *Z* is the degrees of freedom. The statistical importance of the model was verified with adequate precision using Equation (5) and Equation (6). These equations were used to determine the signal-to-noise ratio.

$\text{Adequate precision=}\frac{\text{max }\hat{\text{y}}\text{ - min }\hat{\text{y}}\text{ }}{\sqrt{\overline{\text{V}}\text{(}\hat{\text{y}}\text{)}}}$ (5)

$\overline{\text{V}}\text{（}\hat{\text{y}}\text{）=}\frac{1}{\text{n}}\sum_{\text{i=}1}^{\text{n}} \text{V(}\hat{\text{y}}\text{)=}\frac{p\sigma^{2}}{\text{n}}$ (6)

Which,$\hat{\text{y}}$is the predicted response, *p* is the number of model parameters. $\sigma^{2}$is the residual mean square, and *n* is the number of experiments.

To evaluate the fitting effect of the model on the experimental results, some other important diagnostic plots including Internally Studentized Residuals against Run Number, Predicted against Actual, Internally Studentized Residuals against Predicted and Normal Probability against Internally Studentized Residuals, respectively, were shown in **Figure S1**. All points showed in the Normal Probability against Internally Studentized Residuals plot shown in **Figure S1a** was concentrated in a straight line illustrated that the error was normally distributed. In a plot of Internally Studentized Residuals against Run Number and Internally Studentized Residuals against Predicted, the residuals were randomly distributed between +3.00 and -3.00, indicating that the Box-Behnken model was successfully established the relationship between the independent variable and the reduction efficiency. A plot of Predicted against Actual was shown in **Figure S1b**, the points were approximately distributed on a straight line with a slope of 1, which indicated that this model could accurately predict the actual value.


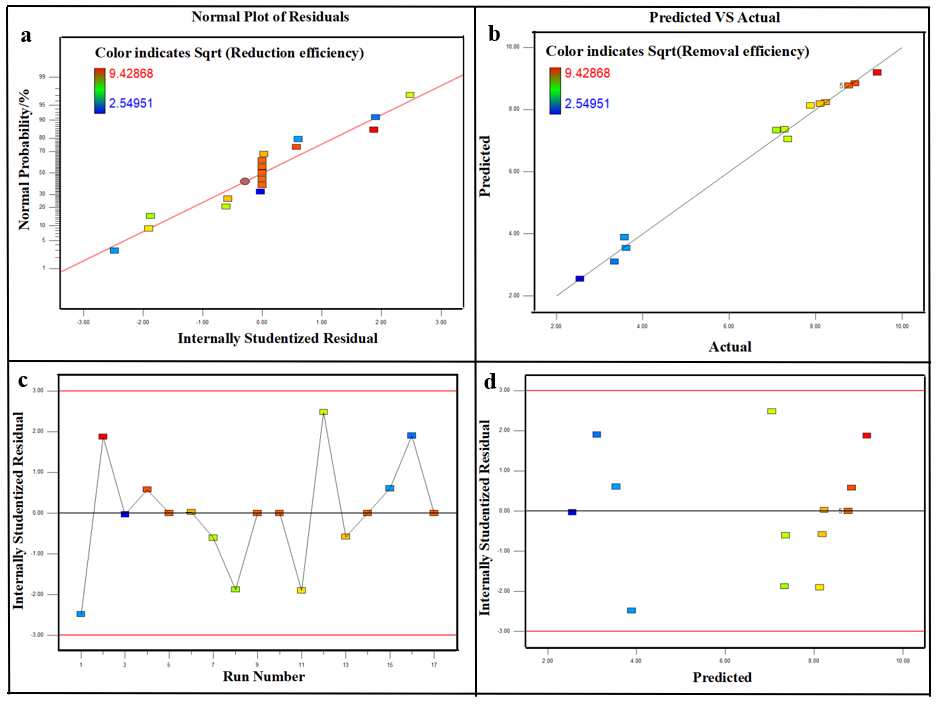


**Figure S1** Diagnostic plots of the quadratic model. (a- Normal Probability against Internally Studentized Residuals; b- Predicted against Actual; c-Internally Studentized Residuals against Run Number; d- Internally Studentized Residuals against Predicted)
